# Supplementary material for: Are carbon emissions trading and green financial instruments synergistic? -Comprehensive quantitative research based on content analysis
Source: PLoS One. 2024 Mar 7;19(3):e0298601. doi: 10.1371/journal.pone.0298601 (PMC10919601; doi:10.1371/journal.pone.0298601)
Supplement: S1 Data — (ZIP) [file pone.0298601.s001.zip › Data/地级市Z_F工作报告.zip!/║╙─╧/╞╜╢Ñ╔╜╩╨/└┤╫╘╣1⁄2╓┌║┼í╛┬φ┐╦╩2╛▌═°í┐_2007.docx]

各位代表：

现在，我代表市人民政府，向大会作政府工作报告，请予审议，并请各位政协委员提出意见。

一、2006年工作回顾

2006年，市人民政府在省委、省政府和市委的正确领导下，深入贯彻科学发展观，积极构建和谐社会，团结奋进，扎实工作，圆满完成了市八届人大四次会议确定的各项任务，实现了“十一五”发展的良好开局。

——经济保持快速增长。初步核算，全市生产总值达到671.9亿元，比上年增长15.8%%。一、二、三产业分别实现增加值73.4亿元、416.1亿元和182.4亿元，增长7.4%%、18.4%%和13.7%%。

——综合效益显著提高。地方财政一般预算收入40.4亿元，增长38.9%%，占生产总值的比重提高0.8个百分点。规模以上工业企业实现利润60.6亿元，增长66.5%%。工业经济效益综合指数182.9%%，提高29.2个百分点。

——“三大需求”明显扩大。全社会固定资产投资272.7亿元，增长49.9%%，其中城镇投资235.8亿元，增长53.9%%。年末金融机构各项贷款余额333亿元，增长32.7%%。社会消费品零售总额168.6亿元，增长15.4%%。外贸出口2.7亿美元，增长29.4%%。

——人民生活继续改善。城镇新增就业11.7万人。城镇居民人均可支配收入9897.5元，农民人均纯收入3150.3元，扣除价格因素，分别增长12.6%%和15.5%%。年末城乡居民储蓄存款余额380.2亿元，增长16.3%%。

去年各项工作的进展主要表现在：

（一）经济转型迈出新步伐。工业结构进一步优化。出台了促进重点工业企业加快发展的意见，制定了鼓励金融部门优化信贷结构、支持地方经济发展的办法。在改造提升煤炭、电力、钢铁、建材、纺织等传统产业的同时，积极谋划和加快化工、机电、食品、医药等新兴接续产业发展，新型能源和工业基地建设迈出坚实步伐。全市规模以上工业中，非煤产业增加值增长30%%；高技术产业增加值增长75.5%%；30户重点企业产品销售收入增长26%%。服务业层次继续提升。传统服务业不断壮大，新兴服务业加快发展。文化及相关产业增加值25亿元，增长16.7%%；旅游总收入15.1亿元，增长42.6%%；房地产开发投资14.9亿元，增长1.3倍，服务业已成为全市经济发展的重要增长点。科技创新能力不断增强。实施国家级火炬计划项目1项，省级计划项目49项，争取省级以上科技经费2523.5万元。新增国家级高新技术企业1家、省级高新技术企业10家。大力支持企业研发中心建设，新增省级工程技术研究中心1家。引导企业实施名牌战略，“神马牌”锦纶工业长丝、“中材环保牌”袋式除尘器荣获“中国名牌产品”称号，新增省名牌产品9个、省优质产品9个。资源节约和环境保护取得明显成效。制定并实施节能降耗、发展循环经济和建设节约型社会的规划，固体废弃物综合利用率提高到67.8%%。继续开展空心村、砖瓦窑厂、工矿废弃地整治，关闭黏土砖瓦窑厂358座，平整、复垦土地342.3公顷。加快淘汰落后产能，关停立窑水泥生产线16条、小珍珠岩厂和煤粉厂8家。对矸石山自燃、扬尘污染、秸秆焚烧等进行了综合整治。城市大气质量达标261天，比上年增加37天。推进天然林保护、退耕还林、速生丰产林基地等重点生态工程建设，完成造林1.1万公顷。

（二）投资结构发生新变化。根据国家新的产业政策和我市实际，在能源、煤盐化工等具有比较优势的领域和农业、水利、交通、社会事业等方面，建成和开工了一批重点项目，发展后劲进一步增强。年初安排的87个重点建设项目，完成投资138.6亿元，增长52%%，占全年预期投资的128%%。其中，瑞平石龙日产2×4500吨水泥、神马集团6000吨高模低缩工业丝、平临高速、市第一人民医院病房楼、市污水处理二期等22个项目建成投运；平煤集团20万吨联碱、平高集团100万伏特高压组合电器、平高东芝二期工程、舞钢及鲁山输变电工程、龙兴寺水库除险加固等12个项目开工建设。新增高速公路通车里程106公里、日污水处理能力17万吨、电力装机53.5万千瓦、新型干法水泥生产能力620万吨。同时，积极清理新开工项目，严肃查处土地违法案件，严格控制高耗能、高污染和产能过剩行业的项目建设，部分行业盲目投资得到遏制。

（三）“三农”工作取得新成效。全年粮食总产182.3万吨，创历史最高水平。农民收入连续两年保持两位数增长，“十五”以来首次超过城镇居民收入增速。对“三农”的投入力度继续加大。全市财政支农支出达到4.3亿元，增长34.8%%。全面落实了对种粮农民直接补贴、良种补贴、大型农机具购置补贴和农资综合补贴等惠农政策。保护价收购小麦57.5万吨，农民直接增收6000万元。提前一年实现了所有行政村通水泥路或油路，新解决8.6万农村人口的安全饮水问题，新增通电自然村28个，新建沼气池3万座。又有4.3万农村贫困人口脱贫。农业产业化经营加快推进。新增农业产业化生产基地1.3万公顷、无公害农产品生产基地24个、无公害畜产品生产基地19个。新增年销售收入100万元以上的农业产业化龙头企业25家。3家企业分别成为全省同行业规模最大或标准化程度最高的生产企业。新增农民专业合作经济组织63个，提高了农民进入市场的组织化程度。农村劳动力转移培训进一步加强。继续实施农村劳动力转移培训“阳光工程”和“农村百万劳动力转移工程”。全年完成农民科技培训41.4万人，农民职业技能培训14.4万人，新转移农村富余劳动力14.3万人，实现劳务收入77亿元。县域经济加快发展。6县（市）生产总值增长17.5%%，地方财政一般预算收入增长53.2%%，分别高出全市1.7和14.3个百分点。4个县（市）财政一般预算收入突破2亿元，其中舞钢市达到6亿元。新农村建设试点工作扎实起步，农村生产生活条件有新的改善。

（四）城镇建设上了新台阶。以创建中国优秀旅游城市为契机，大力推进城镇化进程，全市城镇化率达到37%%，提高2个百分点。老城区建设路、园林路等主次干道改造完成；程平路主体路面改造基本完工；湛河“三桥”及连线建成通车；和平路东段步行街、火车站广场、湛河亮化等工程和28条背街小巷改造相继完成。新城区新开工项目62个，续建项目60个，完成投资36亿元。区内供水、供电、供气、供热等各种管网已铺设到位，已经建成和正在建设的项目建筑面积达400多万平方米，湖滨新城初具规模。实施园林绿化工程，中心城区建成街头游园22个，种植树木228万株，新增绿地263万平方米。同时，以完善体制机制为重点，建立健全各级城管责任制，城市日常管理和环境面貌有了新起色。成功创建中国优秀旅游城市和省级园林城市。县城和小城镇面貌也有明显改观。6县（市）和石龙区全部创建为省级卫生城。舞钢市城乡一体化试点工作进展顺利。

（五）改革开放实现新突破。各项改革深入推进。完成国有企业改制50家，累计达到268家，改制面88.7%%，其中国有工业企业改制面达97.6%%。平煤天安股票成功上市，融资规模居全省上市公司之首。建设系统三项改革全面完成。分配制度改革有序展开。农村综合改革深入推进。行政审批制度改革进一步深化。供销体制、粮食流通体制改革继续巩固完善。其他领域的改革也取得了新进展。对外开放不断扩大。举办了平顶山环渤海（青岛）招商周，组织参加了第四届中国河南国际投资贸易洽谈会、华侨华人创业发展洽谈会等招商引资活动。实际利用外资5809万美元，增长33%%；引进市外资金113.6亿元，增长1.5倍。新批外商投资企业14家，新增进出口经营权企业280家。非公有制经济加快发展。落实中小企业创业辅导和信用担保等扶持政策，大力表彰奖励非公有制经济发展先进单位和个人。新增规模以上非公有制企业438家，非公有制经济实现增加值310.4亿元，增长17.2%%，占全市生产总值的比重提高4.7个百分点。

（六）和谐社会建设有了新进展。认真解决关系人民群众切身利益的问题，向人民群众承诺的“十件实事”全部完成。就业再就业工作继续加强。积极开发就业岗位，发放小额担保贷款5600万元，下岗失业人员再就业4.7万人，均超额实现全年预期目标。解决城镇“零就业”家庭402户，基本消除当年城镇“零就业”家庭。社会保障体系不断完善。城镇企业养老、失业、医疗和工伤保险覆盖面继续扩大，参保人数分别增加1.5万人、1.3万人、0.4万人和2.1万人。进一步提高城市低保补差水平，实现了动态管理下的应保尽保。初步建立了农村低保制度，9.7万人享受农村低保。在6县（市）全面推行了新型农村合作医疗制度，参合率达到76.4%%。全面实施了城乡医疗救助制度，大病救助1860人次。舞钢市在全省率先建立了全民医保制度。爱心超市救助困难群众4万余人次。农村五保对象集中供养率提高到20%%。新建经济适用房18.9万平方米，解决了1764户中低收入家庭的住房困难。新增住房公积金贷款2.8亿元，改善了3280户职工的居住条件。城市居民住房成套率达到86.1%%，提高4.6个百分点。社会事业全面发展。改造中小学校舍8800平方米，基本建立了农村中小学D级危房改造长效机制。资助贫困生43万人次，全面落实了农村义务教育“两免一补”政策。投资4660万元，推进了农村中小学远程教育工程。普通高校在校生达到4万人，增长16.3%%。主办了中国宝丰第三届魔术文化节和全省首届曲剧艺术节。新增国家级重点文物保护单位12处。宝丰马街书会进入首批国家级非物质文化遗产保护名录。解决了边远地区46个自然村（居民20户以上）广播电视“村村通”问题。投资1359万元，完成了20所乡镇卫生院改造。群众体育蓬勃发展，业余训练水平不断提高。省十运会上，我市金牌和积分均列全省第5名。人口和计划生育工作继续保持全省先进。安全生产和社会大局保持稳定。认真执行安全生产的各项法律法规，严格落实责任，强化监督管理，全市各类伤亡事故起数、死亡人数分别下降10.6%%和7.1%%，其他安全生产指标都低于省定控制指标。制定完善了各类应急预案，处理突发事件能力不断增强。实施食品药品放心工程，全年没有发生重大食品药品安全事件。农村消防工作取得新进展，43个乡镇建立了多功能消防队（站）。社会治安防控体系不断完善。市长热线网络建设得到加强。信访秩序逐步好转。精神文明和民主法制建设扎实推进。继续开展精神文明创建活动，推荐省级文明单位38家。深入开展社会主义荣辱观教育，思想道德建设进一步加强。高度重视国防教育，民兵预备役工作取得成效。扎实开展双拥共建活动，军政军民团结更加巩固。自觉接受人大及其常委会的法律监督、工作监督和人民政协的民主监督，125件人大代表建议和287件政协提案全部办理完毕。“五五”普法规划全面启动。认真贯彻行政许可法、公务员法和全面推进依法行政实施纲要，法治、诚信和服务型政府建设不断加强。深入开展勤政廉政建设，加大审计监督力度，治理商业贿赂，反腐倡廉工作取得新成效。民族、宗教、统计、外事、侨务、对台、史志、档案、物价、盐业、地震、人防、气象、新闻出版、国家安全等各项工作都取得了新成绩。

各位代表，过去的一年，是我市现代化建设取得显著成绩的一年。主要发展指标超出预期，并高于全省平均水平，多项总量指标位次稳中有升。特别是在认真执行国家宏观调控政策、不断优化投资结构和财政收入结构的同时，城镇投资增速和重点项目建设综合考评均居全省第1位，财政一般预算收入增速连续两年保持全省第1位。我们深切地体会到，要加快科学发展，必须在科学发展观指导下，走符合自身实际的发展路子；必须积极顺应调控，在调控中加快发展；必须创新发展举措，努力寻求新型工业化、城镇化和农业现代化的新突破；必须坚持以人为本，更多地关注和解决民生问题；必须坚持求真务实，从制度上保证各项工作的落实。

各位代表，这些成绩的取得，是省委、省政府和市委正确领导的结果，是人大、政协积极支持的结果，是全市广大干部群众共同奋斗的结果。在此，我代表市人民政府，向工作在全市各条战线上的工人、农民、知识分子、干部、人民解放军驻平部队指战员、武警官兵和公安民警致以崇高敬意！向关心、支持我市现代化建设的各界人士表示衷心感谢！

在肯定成绩的同时，我们也清醒地看到，我市经济社会发展中还存在一些突出矛盾和问题。主要是：经济增长方式仍然比较粗放，节能降耗和污染减排任务还很艰巨；第三产业发展较慢，占生产总值的比重由上年的27.9%%下降到27.1%%，低于全省平均水平2.2个百分点；农业基础设施薄弱，粮食稳定增产、农民持续增收难度加大；县域经济规模相对较小且发展不平衡，6县（市）生产总值增速最快的比最慢的高10.8个百分点；企业自主创新能力不强，高技术产业增加值占规模以上工业的比重仅为2.3%%，低于全省平均水平1.2个百分点；经济外向度低，外贸依存度5.2%%，低于全省平均水平1.4个百分点；关系人民群众切身利益的不少问题还没有得到很好解决，部分群众生活还比较困难，安全生产还存在薄弱环节，和谐社会建设任重道远；机关作风、行政效能有待继续改进和提高，政府自身建设需要进一步加强。我们必须采取更加有力的措施，切实解决这些问题，决不辜负全市人民的期望！

二、2007年工作总体要求和目标任务

2007年，我们面临的是一个总体上有利于加快发展、但不利因素可能增多的环境。世界经济可望继续保持增长态势，全国经济继续平稳较快增长，国际国内产业转移步伐加快，中部地区正成为新的投资热点，振兴东北老工业基地等优惠政策延伸到我市，为我们加快发展提供了难得机遇。经过多年努力，我市综合实力上了一个新台阶，资源、能源等优势得到进一步发挥，为实现又好又快发展奠定了良好基础。特别是市七次党代会提出的在科学发展观指引下“走在全省前列、率先实现崛起”的宏伟目标，极大鼓舞着全市人民干事创业的热情。同时，国家宏观调控力度加大，用地审批更严、信贷控制更紧、市场准入“门槛”更高，为我们转变增长方式、推进经济转型既带来了更大的压力，也提供了新的动力。我们要以更加饱满的精神状态，更加务实的工作作风，创造性地开展工作，努力把各项工作做得更好。

今年政府工作的总体要求是：以邓小平理论和“三个代表”重要思想为指导，全面落实科学发展观，扎实构建社会主义和谐社会，认真贯彻党的十六大以来各项方针政策和省八次党代会、市七次党代会精神，紧紧围绕“走在全省前列、率先实现崛起”的战略目标，按照又好又快发展的要求，更加注重调整经济结构和转变增长方式，更加注重节能降耗和环境保护，更加注重改革开放和自主创新，更加注重促进社会发展和解决民生问题，全面推进经济建设、政治建设、文化建设和社会建设，统筹做好改革发展稳定的各项工作，迈出建设实力、高效、文化、和谐、魅力平顶山的坚实步伐。

按照这一要求，综合考虑各方面因素，今年经济社会发展的预期目标是：生产总值增长12%%以上；地方财政一般预算收入增长13%%以上；全社会固定资产投资增长18%%以上；社会消费品零售总额增长15%%以上；外贸出口增长15%%以上，实际利用外资增长20%%以上；城镇居民人均可支配收入增长11%%左右，农民人均纯收入增长8%%左右；居民消费价格上涨3%%以内；万元生产总值能耗降低4.5%%；化学需氧量、二氧化硫排放量分别减少10%%和3.7%%；城镇新增就业6.5万人，城镇登记失业率控制在4.5%%以内；人口自然增长率控制在6.5‰以内。

在完成上述目标的同时，我们将积极筹措资金，着力办好事关群众切身利益的“十大实事”：一是加大对农民的补贴力度，确保补贴金额高于上年；二是改善农民基本生产生活条件，繁荣农村文化事业；三是巩固提高农村义务教育水平，建立高中阶段贫困学生救助制度；四是建立全民医保制度，缓解群众看病难、吃药贵问题；五是加大农民培训力度，做好农村劳动力转移就业工作；六是加强城镇就业再就业工作，完善社会保障体系；七是加大对困难群体的救助力度，完善城乡救助体系；八是进一步加强城市基础设施建设，提升城市服务功能；九是改善城市居民住房条件，加大对矿区棚户区改造力度；十是进一步治理环境污染，改善生态条件。使改革发展的成果更多地体现在全市人民能够享受的实惠上。

三、努力促进经济又好又快发展

坚持科学发展，立足加快发展，把经济工作的重点放到优化结构、提高效益和降低消耗、保护环境上来，增强经济竞争力和可持续发展能力。

（一）顺应宏观调控，进一步加强重点项目建设。当前，投资仍是拉动我市经济增长的主要力量。要认真贯彻国家宏观调控政策，坚持区别对待、有保有压，优化投资结构，保持适度的投资规模。今年城镇固定资产投资计划完成281亿元，增长19%%。安排重点项目89个，年度投资110亿元。其中，工业项目56个，年度投资52.8亿元。年内建成平煤集团瑞平公司2×15万千瓦热电联产机组，神马集团尼龙66切片连续聚合技改、双30万吨氯碱、500吨赛尔纤维，舞钢公司100万吨宽厚板生产线，姚电四期2×60万千瓦一号机组，平高东芝二期扩建，海明集团5万吨改性木质素等26个项目；开工建设平煤集团30万吨PVC及配套25万吨烧碱、平西50万伏超高压输变电工程等7个项目；加快建设平煤天安十一矿改扩建、神马集团20万吨尼龙66盐成套、平高集团100万伏超高压组合电器、圣光公司药械基地等12个续建项目。农业、交通、城建等基础设施项目26个，年度投资56.2亿元。年内建成农村安全饮水、郑石高速平顶山段、各县（市）和石龙区垃圾处理厂等5个项目；开工建设孤石滩灌区续建配套、焦桐高速叶舞段等9个项目；加快建设燕山水库、太澳高速平顶山段等8个续建项目。社会事业项目7个，年度投资9655万元。年内建成平煤总医院综合楼、新城区湖光小学等项目；加快建设平顶山工学院西校区二期工程。做好后续项目的前期工作，力争平煤集团80万吨联碱、姚电五期2×100万千瓦机组、叶县蓝光电厂二期2×30万千瓦循环流化床机组等项目早日立项并批准建设。继续坚持项目建设各项制度，创造环境，落实资金，紧盯进度，及时解决工程进展中的困难和问题。同时，积极培育和扩大消费需求，拉动经济快速健康发展。

（二）推进经济转型，加快建设新型能源和工业基地。积极研究、充分利用国家扶持老工业基地发展的优惠政策，促进经济转型。全市规模以上工业增加值增长18%%以上，利润增长12%%以上。继续引导工业结构优化。加快用先进适用技术改造提升传统产业。切实用好市工业结构调整资金、金融信贷支持等优惠政策，引导煤炭、电力、钢铁、建材、纺织等传统产业的骨干企业，加大技术改造力度，加快信息化建设步伐，提高技术装备水平和生产能力。同时，发挥比较优势，拉长产业链条，做强煤——电产业链，做大煤——化、盐——化产业链，完善采矿——冶铁——炼钢——轧钢产业链。新增电力装机100万千瓦、尼龙66切片3万吨、离子膜烧碱30万吨、PVC树脂30万吨、钢及钢板100万吨，初加工产品比重大幅下降，帘子布、高压电器、电收尘器、铁路重载合金钢铸件等主要产品在全国乃至世界的竞争优势明显增强。大力发展高新技术产业。全年推荐申报高新技术企业3家、高新技术产品5项，高技术产业增加值占规模以上工业增加值的比重提高到2.4%%。切实加快产业聚集区建设。综合运用土地供给、环境保护、金融信贷、资源配置等政策，引导工业企业向产业聚集区集中。中心城区化工产业聚集区上半年要完成控制性详细规划，年内开工建设入区道路、供水、供电和污水处理等基础设施项目。各县（市、区）工业聚集区也要尽快完善基础设施，加快项目建设，形成产业规模。积极扶持重点企业发展。全面落实促进重点工业企业加快发展的意见，落实金融支持、结构调整、名牌战略等配套政策，做大做强重点工业企业。鼓励平煤、神马、舞钢、姚电、平高、天瑞、汇源、中加、洁石等重点骨干企业，通过资产重组、联合、并购等手段，优化生产要素和资源配置。加快企业上市步伐。加大宣传、培训和辅导力度，增强企业上市意识；支持天瑞集团整合优势资产，做好上市前期工作；扶持中加、巨龙、银龙等优势企业加快发展，创造条件上市。通过努力，30户重点工业企业年销售收入突破600亿元，其中平煤集团超300亿元，神马集团、舞钢公司分别超100亿元。着力增强企业自主创新能力。加强企业研发中心建设，新建市级以上工程技术中心4个。做好科技成果鉴定和推广工作，完成新成果鉴定80项，转化推广成熟技术16项。争取再创省以上名牌产品3~5个。实施知识产权推进工程，专利申请量达到200项以上。加强产学研结合，依靠科技进步，推动经济增长由资源依赖型向创新驱动型转变。

（三）发展现代农业，扎实推进社会主义新农村建设。认真落实支农惠农政策。继续实施对种粮农民直接补贴、农业生产资料综合直补、优质专用良种补贴、测土配方施肥补贴、农村劳动力转移培训补贴和大型农机具购置补贴。进一步加大“三农”投入力度，确保财政支农支出的增量继续高于上年，固定资产投资用于农村的增量继续高于上年，土地出让收入用于农村建设的增量继续高于上年。继续实行粮食最低收购价政策，保持粮食和农业生产资料价格基本稳定。加快推进农业结构调整。在稳定粮食生产的前提下，大力发展高效种植业，新增优质小麦、优质玉米和优质油料、烟叶、蔬菜、瓜果等经济作物种植面积1.16万公顷，培育无公害农产品生产基地20个。重点发展畜牧业，加快推进优质畜产品生产基地和奶源基地建设，新建规模养殖场200个、无公害畜产品生产基地15个、奶牛规模养殖小区50个。积极发展林果业，新增速生丰产林、名特优经济林、生物质能源林3500公顷。完成鲁山县、石龙区省级林业生态县创建任务。努力提高农业产业化经营水平。市农业结构调整资金重点用于扶持龙头企业发展，着力培育一批在省内外有较高知名度的特色农产品品牌。新增年销售收入100万元以上的农业产业化龙头企业28家。健全农业社会化服务体系。推进农产品质量安全监测和认证体系建设，推行农产品市场准入制度。加强农业信息服务体系建设，开通农业信息综合服务热线。加快畜牧兽医管理体制改革，完善基层动物防疫体系。积极发展农业合作经济，新增农民专业合作经济组织50家，带动更多的农户进行专业化、标准化、规模化生产和经营。大力发展劳务经济。继续实施“农村百万劳动力转移工程”，加大农村劳动力培训力度，与发展职业教育相结合，提高培训水平，打造劳务品牌。完成农民科技和技能培训54万人，新增农村劳动力转移就业10万人。鼓励外出务工人员回乡创业。继续加强农村基础设施建设。再解决10万以上农村人口的安全饮水问题，完成650公里农村公路建设改造任务，解决农村用电盲点户1.9万户，新增农机总动力5万千瓦，新建沼气池4万座。健全农村市场体系，积极发展农村超市、连锁店和农家店。高度重视并妥善做好移民安置工作。加大扶贫开发力度，全年脱贫4万人。进一步壮大县域经济。用足用活国家和省促进县域经济发展的扶持政策，加强与上级有关部门的沟通衔接，抓好资金和项目的落实。支持经济强县（市）率先发展，扶持困难县加快发展，努力提高县域工业增加值、非公有制经济增加值和财政收入占生产总值的比重，促进各县（市）在全省综合经济实力排序中升级晋位。继续搞好新农村建设试点，及时总结试点村经验，指导新农村建设分类型地整体推进。

（四）创建国家园林城市和卫生城市，大力提升城镇化水平。坚持中心城市带动战略，加快县城和小城镇建设，力争城镇化率提高2个百分点。进一步加大中心城区建设和管理力度。在巩固创优成果的基础上，全面开展国家园林城市、卫生城市创建工作，通过今明两年的努力，实现创建目标。坚持规划先行，认真做好规划编制、审批和执法工作，切实发挥规划的龙头带动作用。老城区要完成新华路北延、开源路南延、凌云路等跨年度工程和高阳路三期工程，完成新华路、开源路、程平路等路段的绿化改造及城郊防护林建设任务。开工新建28个街头游园。加快推进建西采煤塌陷地综合整治。继续搞好湛河治理。加快工人文化宫升级改造、联盟路连片开发及和平路西段商业步行街改造步伐。继续推进“城中村”和背街小巷改造。加大环卫设施建设力度，筹备建设垃圾无害化处理厂，建成市医疗废物处置中心。积极发展城市公交，再更新大巴车100台、出租车100辆。新城区建设用地框架要拉大到25平方公里。年内完成经九路、经十二路、湖滨东段景观绿化等工程。加快纬四路改造、湖滨西段景观绿化、第七中学等续建项目建设。开工建设纬六路、第三安置小区和平煤沉陷区安置小区等项目。加快行政入区项目进度，2005年开工的项目，必须投用；2006年开工的项目，争取投用。进一步理顺城市管理体制，实施城市精细化管理，全面提高城市管理水平。加强市政设施维护，保证市政设施功能完好。继续加快县城和小城镇建设步伐。今年各县（市、区）要完成建成区内所有背街小巷、居住区、市场等整治任务。以31个重点镇建设为重点，指导乡镇政府完善乡镇规划，搞好道路硬化、绿化和农贸市场建设，增强小城镇的吸纳承载能力。年内每个县（市）要有1~2个小城镇建设通过达标验收。舞钢市城乡一体化试点工作要有实质性进展。大力发展和提升服务业。以旅游开发为龙头，带动第三产业全面发展。加快景区建设，启动石人山创5A级景区工作，力争再有2~3家景区进入4A级行列。加大温泉资源开发力度。加强旅游宣传推介，培育旅游企业集团，打造知名旅游品牌。全市旅游接待人数和旅游总收入均增长15%%以上。支持流通业态创新，加快运用现代服务技术和经营方式，改造提升商贸、餐饮等行业。大力发展现代物流，加强物流市场体系建设。积极促进金融、保险、房地产、信息、商务中介等现代服务业发展。

（五）加强节能减排，努力建设资源节约型和环境友好型社会。强力推进节能降耗。建立健全节能降耗目标责任制，完善能耗指标公报和重点用能单位能源利用报告制度。大力倡导节约型生产方式和消费方式。加快先进节能技术、节能产品的研发和推广应用。着力抓好冶金、煤炭、电力、化工、建材等高耗能行业和重点耗能企业的节能降耗。综合运用产业政策、环境容量、差别电价等措施，加快淘汰落后工艺、设备和生产能力。积极推进建筑、交通、商业等领域的节能降耗。大力发展循环经济。加强再生资源回收。支持各类企业搞好循环生产，充分利用工业废水、废气、废渣，变废为宝，最大限度提高资源利用效率。支持发展煤矸石（煤泥）电厂、粉煤灰综合利用等项目，做大做强煤矸石烧结砖产业，提高市场占有率。固体废弃物综合利用率达到70%%以上。进一步提高土地集约利用水平。加强耕地特别是基本农田保护。从严控制新增建设用地规模，严格执行新的土地有偿使用费标准，严格实行土地出让“招拍挂”制度。以16个工业聚集区为重点，加快推广使用多层标准厂房，提高单位土地面积投资强度和投资效益，积极追求同行业亩均产值效益最大化。巩固黏土砖瓦窑厂关闭整顿成果，完成土地整理和复垦374公顷。继续整顿和规范矿产资源开发秩序。切实加强环境保护。今年要削减化学需氧量3400吨、二氧化硫排放量5000吨，城区大气环境质量达标261天以上，城市集中饮用水源地水质达到或优于地表水3类标准，出境水质基本达标。要认真落实环境目标责任制，严格执行环境影响评价、污染物排放总量控制和项目建设“三同时”等制度。深化环保专项整治，年内关闭所有立窑水泥生产线和市规划区内的所有小煤粉厂、小珍珠岩厂；关闭6家12台5万千瓦以下小火电机组，完成4家电厂（站）脱硫设施配套建设任务。巩固城区周边环境治理成果，矸石总量不再增加。强化环保执法监督，对重点污染源安装在线监控设施，严厉查处环境违法案件，确保环境质量进一步改善。

（六）深化改革开放，不断增强发展动力和活力。继续深化各项改革。全面推进中小型工业、商贸、建筑、粮食、供销、物资企业改革，已进入改制程序但未完成改制的4家工业企业和18家非工业企业，年内基本完成改制重组；具备条件但未进入改制程序的企业，加快进入改制程序；指导帮助已改制企业解决发展过程中存在的困难和问题，巩固改革成果。稳步推进大中型企业主辅分离、辅业改制工作，全面完成企业办学校移交任务。加快经营性事业单位改制步伐，年内完成市接待单位的改制任务。巩固乡镇机构改革成果，把精简机构、分流人员措施落到实处。加快县乡财政体制改革，完善“乡财县管”制度。开展清理化解乡村债务试点工作。实施农村公益事业“一事一议”筹资筹劳办法。积极探索土地承包经营权依法流转新路子，发展适度规模经营。加快林权制度改革，推进林地、林木经营权和所有权的合理流转。支持城市信用社组建城市商业银行。积极推进农村信用社改革。全面推行煤炭资源有偿使用制度，建立健全其他重要矿产资源有偿使用和生态补偿机制。完成国省干线公路建设管理体制改革和农村公路养护体制改革。深化收入分配制度改革，统筹安排预算内、外财力，确保工资和津贴、补贴发放。加快发展开放型经济。创新招商引资方式，构建社会化、市场化、专业化的招商网络。积极组团参加第二届“中博会”、第十一届“投洽会”、“华创会”等重大招商活动，推动优势产业和基础设施项目开展对外合资合作。引导支持各类产业聚集区充分发挥政策、区位、产业配套等优势，积极承接产业转移。加大对县域利用外资工作的指导和督查，改变利用外资水平较低的状况。优化出口商品结构，鼓励和扩大农副产品出口，扶持高压开关、电收尘器、碳化硅等机电产品和高新技术产品出口。加快发展对外承包工程与劳务合作，不断扩大外派劳务规模。进一步壮大非公有制经济。优化发展环境，提高服务质量，落实有关政策措施，依法保护非公有制经济的合法权益。着力帮助中小企业解决贷款难问题，上半年各县（市、区）贷款担保中心全部注入资本金并正常运作。支持引导非公有资本进入现代服务业、高新技术产业和社会事业等领域。力争非公有制经济占生产总值比重和对财政收入的贡献率均达到50%%以上。

四、切实加强文化建设

认真落实徐光春书记对“宝丰现象”的批示精神，深入开展“拓展宝丰现象、建设文化鹰城”活动，提升城市文化品位，提高社会文明程度。

（一）大力繁荣文化事业。加大公益性文化基础设施建设投入。市博物馆、文化艺术中心主体工程年内基本完工。加强县级文化馆、图书馆、乡镇文化站和新农村书屋建设，年内建成舞钢市文化活动中心和鲁山县宣传文化中心。继续实施文化信息资源共享、农村电影放映、农村广播电视“村村通”等工程。实施文化精品工程，支持市文艺团体申报中国舞台艺术精品剧目。进一步繁荣发展新闻出版、广播影视、文学艺术和哲学社会科学。充分发挥戏曲文化优势，为每个县级剧团各配备一个流动舞台，活跃城乡文化生活。办好首届社区文化艺术节和平顶山特色文化活动周。加强重点文物和非物质文化遗产保护，启动宝丰清凉寺汝官窑遗址申报世界文化遗产工作。

（二）加快发展文化产业。充分发挥我市佛教文化、姓氏文化、名人文化、古县衙文化、红色文化、知青文化等资源优势，加大开发和宣传力度，打造我市文化名片。大力发展陶瓷文化产业。成立市汝瓷研究会，加大汝瓷的研发、生产和宣传力度，既要注重规模效益，更要注重质量和文化。着力发展民间演艺文化产业。研究马街书会的文化内涵，创新举办形式，提高知名度和影响力。进一步提高宝丰魔术的演艺水平，重点扶持具有一定规模和市场竞争力的民间团体、精品节目和道具生产企业，带动我市群众演艺业走向全国、走向世界。加快文化产业基地和特色文化产业园区建设，做好省级文化产业示范基地申报工作。积极推动文化产业和高新技术产业相结合，加快有线电视向数字化转换。加强文化产业项目库建设，编制《平顶山市文化产业项目投资指南》。积极开展对外文化交流合作，大力培育外向型文化企业。强化文化市场监管，深入开展“扫黄打非”和反盗版斗争。

（三）积极推动文化体制改革。创新管理体制，实行管办分离，实现政府职能由办文化向管文化、由管微观向管宏观转变。深化文化事业单位内部人事、收入分配和社会保障制度改革。积极推进经营性文化事业单位转企改制。政府除对重点艺术院团等少数公益性文化事业单位给予扶持外，一般性演出团体和演出场所都要推向市场。鼓励和支持民营企业参与国有文化单位改革，引导非公有资本进入文艺表演、文化旅游、书报音像制品分销等文化产业领域，积极扶持民间职业演出团体和农村业余剧团，促进民营文化企业健康发展。

（四）进一步加强精神文明建设。把建设社会主义核心价值体系融入国民教育和精神文明建设全过程，深入开展以“八荣八耻”为主要内容的社会主义荣辱观教育，切实加强社会公德、职业道德、家庭美德教育，广泛开展群众性精神文明创建活动。加强未成年人思想道德建设和大学生思想政治教育工作。积极开展学习型机关和创新型机关创建活动，全面推动机关文化、企业文化、社区文化和校园文化建设。进一步组织好文化、科技、卫生“三下乡”和科技、文体、法律、卫生进社区活动，提高全民科学文化素质。加快信用体系建设，在全社会营造诚信为本、守信光荣的良好风气。

五、扎实构建和谐社会

坚持以人为本，把促进社会发展和解决民生问题作为和谐社会建设的重点，切实解决好人民群众最关心、最直接、最现实的利益问题。

（一）努力扩大就业。落实各项促进就业政策，多渠道开发就业岗位，确保实现城镇新增就业目标。加大再就业扶持力度，启动再就业孵化园区建设，实现下岗失业人员再就业2.5万人，其中“4050”人员9000人，发放小额担保贷款5300万元以上。继续消除城镇“零就业”家庭。加大技能人才培养力度，进一步推进素质就业。高度重视高校毕业生和退伍转业军人的就业问题，引导各类毕业生进入人才市场择业，鼓励更多符合条件的军转干部自主择业。积极探索建立有劳动能力的城市低保对象就业与低保相互衔接的联动机制，提高就业率。合理调整和严格执行最低工资制度，落实小时最低工资标准，加强企业工资分配调控和指导，建立健全工资正常增长机制和支付机制。发展和谐劳动关系，劳动合同签订率90%%以上。

（二）不断完善社会保障体系。扩大城镇社会保险覆盖面，尽快使养老、医疗、失业、工伤和生育等社会保险覆盖到各类企业。切实关注企业离退休人员养老金发放问题，按时足额发放率达到100%%。积极推行适合农民工特点的工伤、医疗、养老等社会保障制度。继续加大农民工工资清欠力度，确保不发生新的拖欠。健全城市居民最低生活保障制度，实现动态管理下的应保尽保。完善农村居民最低生活保障制度，低保对象增加到12.1万人，每人每月补差不低于30元。农村五保对象集中供养率达到30%%。加快经济适用房建设，开工改造棚户区80万平方米。扩大廉租住房覆盖面，对城镇低收入无房家庭发放住房租赁补贴。城镇职工住房公积金扩面两万人，发放公积金贷款3亿元。市区居民住房成套率达到87%%以上。积极发展社会福利和慈善事业，鼓励引导企业和个人捐赠支持社会公益活动，进一步办好爱心超市。强化社保基金、住房公积金等社会公共基金监督管理，严禁侵占挪用。

（三）大力发展社会事业。优先发展教育事业。巩固提高义务教育水平，实现“两基”国检达标。扩大高中阶段教育规模，普通高中和中等职业学校在校生分别增长8%%和10%%。支持高等教育办出特色、提高水平。鼓励发展民办教育。加强教师队伍建设，积极推行素质教育，提高教育教学质量。深化农村义务教育改革，确保教育投入按时足额到位。坚持公共教育资源向农村和边远地区倾斜，促进教育均衡发展。全部免除农村义务教育阶段学生的学杂费，继续对困难家庭学生免费提供课本和补助寄宿生生活费。保障农民工子女平等接受义务教育。建立高中阶段贫困学生救助制度，继续对高校贫困学生进行资助。筹措资金2695万元，再完成7.6万平方米的校舍维修改造任务。继续推进农村中小学远程教育工程。从今年起，用两年时间把贫困地区农村中小学课桌凳更新配置一遍。规范学校收费行为，严禁教育乱收费。进一步完善培养、引进和使用人才的制度措施，为科学发展提供人才保障和智力支持。加快发展医疗卫生事业。建好医疗服务和医疗保障两个体系，为群众提供安全、优质、方便、价廉的基本医疗服务。加强卫生基础设施建设，筹措资金2.18亿元，再改造3所市医院、5所县医院和24所乡镇卫生院。加快社区卫生服务机构和村卫生所（室）规范化建设。在去年建立新型农村合作医疗制度的基础上，今年建立城镇居民合作医疗制度。完善城乡医疗救助制度，探索建立大病、急病困难患者和无主病人就医保障制度。规范医疗器械和药品招标采购，认真治理医疗乱收费，降低药品和医疗服务价格。积极发展体育事业。加强城乡基层体育设施建设，大力开展全民健身活动，不断提高业余训练水平。认真做好全国第六届城市运动会组团参赛工作，筹办好市七运会。稳定低生育水平，提高出生人口素质，统筹解决出生人口性别比偏高、流动人口管理与服务、人口老龄化、残疾预防等问题。

（四）切实加强安全生产监督管理。全面落实安全生产责任制，突出抓好煤矿安全生产管理，打好瓦斯治理和水害防治攻坚战。继续深化非煤矿山、危险化学品、民爆物品、交通、消防等重点领域的安全专项整治，加强学校和人员密集场所安全防范。积极推进农村和城市社区安全工作，逐步建立农村和城市社区安全管理网络。依法落实企业安全生产主体责任和部门监管责任，严格责任追究，坚决遏制重特大事故发生，确保完成各项安全生产控制指标。大力整顿和规范市场经济秩序，切实加强食品药品安全监管，确保人民群众饮食和用药安全。

（五）全力维护社会稳定。扎实推进平安建设，建立健全社会治安防控体系，深入开展命案侦破、打黑除恶等专项斗争，严厉打击“两抢一盗”等各类违法犯罪活动。全面推进城市社区建设，健全新型社区管理和服务体制，完善中心城区71个社区的基础设施，增强社区综合服务功能。积极开展农村社区建设试点。加强应急体系建设，筹建与国家、省联网的应急指挥平台，提高应对各类突发公共事件的能力。落实信访工作责任制，加强基层调解工作，及时排查纠纷、化解矛盾，引导群众依法有序上访，维护社会大局稳定。

深入开展国防教育，增强全民国防意识。支持驻平解放军和武警部队建设，抓好人民防空和民兵预备役工作。认真落实军人优待抚恤政策，巩固扩大双拥成果。充分发挥工会、共青团、妇联等人民团体的桥梁纽带作用。全面贯彻党的民族宗教政策，依法管理宗教事务。落实“五五”普法规划。搞好基层司法所建设。加强法律援助，依法保障公民特别是妇女、未成年人、残疾人和农民工的合法权益。加强统计监督，提高服务质量。继续做好外事、侨务、对台、史志、档案、物价、盐业、地震、气象、保险、通信、国家安全等工作。

六、加快推进政府自身改革和建设

完成今年的工作任务，必须切实加强政府自身建设。要以转变政府职能为核心，大力推进体制和管理创新，加快建设法治、诚信和服务型政府，增强政府执行力和公信力。

（一）进一步转变政府职能。在搞好经济调节和市场监管的同时，切实加强社会管理和公共服务职能，更多地关注社会和公共服务领域，更好地为市场主体服务和创造良好环境，提高社会管理水平和公共服务质量。继续推进政企分开，政府及各部门不得包办、干预企业投资决策和正常的生产经营活动。积极促进行业协会和中介机构健康发展。继续深化行政审批制度改革，进一步减少和规范行政审批事项，简化办事程序，提高办事效率，办好行政审批服务中心。不断深化政务公开，确保权力公开透明运行。全面推行学校、医院、供水、供电、供气、供热等公用事业单位办事公开制度，保障人民群众的知情权、参与权和监督权。

（二）加快推进管理创新。创新行政管理方式，健全科学民主依法决策机制，落实市政府工作规则，坚持重大事项集体研究决定。完善重大事项专家论证咨询、社会公示与听证、决策评估等制度，提高决策的民主化、科学化和制度化水平。建立健全符合科学发展观要求的经济社会发展综合评价体系和考核指标体系，强化政府绩效评估，建立行政问责制度，确保政令畅通。加强财政性资金审批、监管，提高财政精细化管理水平。创新社会稳定保障机制，进一步建立和完善利益协调机制、诉求表达机制、矛盾调处机制和权益保障机制，坚决纠正土地征用、房屋拆迁、企业改制、环境保护中损害群众利益的行为。加快电子政务建设，提高行政效率。加强市长热线网络建设，切实解决人民群众反映的热点难点问题。

（三）全面推进依法行政。认真落实全面推进依法行政实施纲要，严格依照法定权限和程序行使权力、履行职责、接受监督。规范行政行为，完善执法程序，进一步减少行政执法层级，推行相对集中行政处罚权制度和综合执法试点。规范行政处罚自由裁量行为，促进公正、公平、文明执法。认真落实行政执法责任制和执法责任追究制，严肃查处失职渎职和滥用职权行为。加强行政层级监督、监察监督、审计监督和社会舆论监督，完善行政复议、行政赔偿和行政补偿制度，确保公共行政权力依法、公正、有效运行。认真贯彻监督法，全面执行人大及其常委会的决议，自觉接受人大监督。主动加强与人民政协的联系，认真听取各民主党派、工商联、无党派人士和人民团体的意见。坚持与人大代表、政协委员的联系制度，及时、高质量地办理议案、建议和提案。

（四）切实加强政风建设。认真学习胡锦涛总书记在中纪委第七次全会上的讲话精神，深入开展“讲正气、树新风”主题教育活动，全面加强思想作风、学风、工作作风、领导作风和生活作风建设，大力倡导“八个方面”的良好风气。只争朝夕、扎实精细地推动工作落实，坚决克服办事拖拉、推诿扯皮等不良作风。切实改进会风、文风，进一步清理评比、达标、表彰活动，力戒形式主义和官僚主义。牢记宗旨，心系群众，完善为民服务长效机制。严格规范公务接待行为，严格公费出国出境管理，厉行节约，勤俭办事，反对铺张浪费。加大反腐倡廉工作力度，完善教育、制度、监督并重的惩治和预防腐败体系。深入推进治理商业贿赂工作，严厉打击工程建设、土地出让、产权交易、医药购销、政府采购、资源开发等重点领域的腐败行为。加强对公务员的教育、管理和监督，努力建设一支政治坚定、业务精通、清正廉洁、作风优良、人民群众满意的公务员队伍。

各位代表，做好今年的政府工作，对于落实市七次党代会提出的“走在全省前列、率先实现崛起”目标意义重大。让我们紧密团结在以胡锦涛同志为总书记的党中央周围，同心协力，锐意进取，努力开创我市经济社会发展新局面，以优异成绩迎接党的十七大胜利召开！
